# Abnormal centrosome and spindle morphology in a patient with autosomal recessive primary microcephaly type 2 due to compound heterozygous *WDR62* gene mutation

**DOI:** 10.1186/1750-1172-8-178

**Published:** 2013-11-14

**Authors:** Heba Gamal Farag, Sebastian Froehler, Konrad Oexle, Ethiraj Ravindran, Detlev Schindler, Timo Staab, Angela Huebner, Nadine Kraemer, Wei Chen, Angela M Kaindl

**Affiliations:** 1 Institute of Cell Biology and Neurobiology, Charité University Medicine Berlin, Campus Virchow-Klinikum, Augustenburger Platz 1, Berlin 13353, Germany; 2 Department of Pediatric Neurology, Charité University Medicine Berlin, Berlin, Germany; 3 Berlin Institute for Medical Systems Biology, Max Delbrück Center for Molecular Medicine, Robert-Rössle-Str. 10, Berlin 13092, Germany; 4 Institute for Human Genetics, Technical University Munich, Munich, Germany; 5 Institute for Human Genetics, University of Würzburg, Würzburg, Germany; 6 Department of Pediatrics, University Hospital, Technische Universität Dresden, Dresden, Germany

**Keywords:** Microcephaly, *WDR62* mutation, Cell division, Intellectual disability

## Abstract

**Background:**

Autosomal recessive primary microcephaly (MCPH) is a rare neurodevelopmental disease with severe microcephaly at birth due to a pronounced reduction in brain volume and intellectual disability. Biallelic mutations in the WD repeat-containing protein 62 gene *WDR62* are the genetic cause of MCPH2. However, the exact underlying pathomechanism of MCPH2 remains to be clarified.

**Methods/results:**

We characterized the clinical, radiological, and cellular features that add to the human MCPH2 phenotype. Exome sequencing followed by Sanger sequencing in a German family with two affected daughters with primary microcephaly revealed in the index patient the compound heterozygous mutations c.1313G>A (p.R438H) / c.2864-2867delACAG (p.D955Afs*112) of *WDR62*, the second of which is novel. Radiological examination displayed small frontal lobes, corpus callosum hypoplasia, simplified hippocampal gyration, and cerebellar hypoplasia. We investigated the cellular phenotype in patient-derived lymphoblastoid cells and compared it with that of healthy female controls. WDR62 expression in the patient’s immortalized lymphocytes was deranged, and mitotic spindle defects as well as abnormal centrosomal protein localization were apparent.

**Conclusion:**

We propose that a disruption of centrosome integrity and/or spindle organization may play an important role in the development of microcephaly in MCPH2.

## Introduction

**Figure 1:**
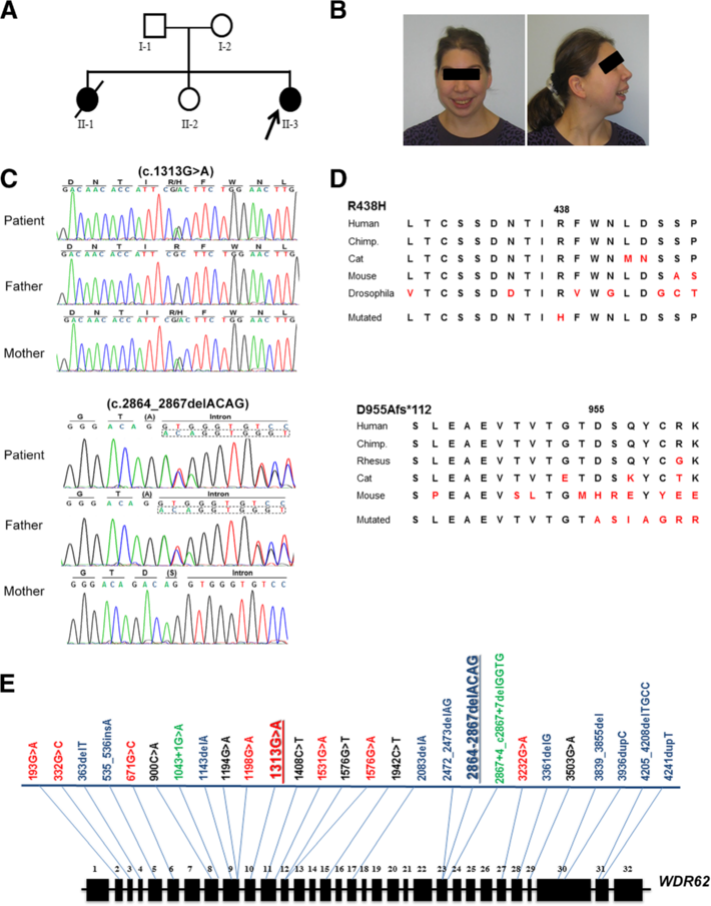
**Phenotype and genotype of index patient. (A)** Pedigree. **(B)** Facial phenotype of the patient. Note the sloping forehead, the convex facial profile, the full lips, and the small chin. The appearance of low-set and posteriorly rotated ears on the lateral picture is partly due to reclination of the head. See Additional file 1: Figure S1 for photo of sibling II:1. **(C)** Representative electropherogram traces confirm the heterozygous frameshift mutation c.2864-2867delACAG in the index patient and her father and the heterozygous missense mutation c.1313G>A in the index patient and her mother (NM_001083961.1 transcript reference sequence). **(D)** Sequence alignment of corresponding WDR62 protein regions depict the highly conserved amino acids affected by the maternally inherited missense mutation (p.R438H) and the position of the paternally inherited frameshift mutation (p.D955Afs*112): Human (Homo sapiens) UniProt O43379, Chimpanzee (Pan troglodytes) GenBank JAA38944.1, Rhesus monkey (Macaca mulatta) GenBank AFH29290.1, Cat (Felis catus) GenBank XP_003998018.1, Mouse (Mus musculus) GenBank NP_666298.3, Fruit fly (Drosophila melanogaster) Fly Base ID FBgn0031374. **(E)** Known *WDR62* gene mutations according to HGMD Professional 2012.4 and the present paper. Mutations types are color-coded, i.e. missense in red, frameshift in blue, nonsense in black, and splice site mutations in green. The positions of the mutations detected in the index patient are emphasized through bold letters (c.1313G>A also present in the index patient has been previously reported [4]).

Autosomal recessive primary microcephaly (MCPH) is a rare neurodevelopmental disorder that results in severe microcephaly at birth with reduction in brain volume, simplified neocortical gyration, and intellectual disability [1-3]. Biallelic mutations in the WD repeat-containing protein 62 gene *WDR62* cause MCPH2 (MIM#604317), the second most common MCPH subtype [4]. So far, 25 mutations of the *WDR62* gene have been reported in 27 families or individual patients worldwide, most of them predicted to produce truncated proteins [4-12] (Figure 1, Table 1). Despite the classic MCPH definition of an isolated microcephaly at birth without severe architectonical abnormalities of the brain, patients with *WDR62* mut.ations can display a wide spectrum of cortical malformations including cortical thickening, polymicrogyria, simplified gyral pattern, pachygyria, schizencephaly, heterotopias, and corpus callosum abnormalities. Some patients also have evidence of lissencephaly, cerebellar hypoplasia, and hippocampal dysmorphy [4,7,12] (Table 1).

**Table 1:** MCPH2 phenotypes and genotypes

**Exon/Intron**	**Mutation**	**Alteration**	**Ethnicity**	**No. of patients**	Mc* at birth	Mc* at follow up	**Neurological phenotype**						**Neuroradiological phenotype**							**Further phenotypes**	**Reference**
							**Intellectual disability**	**Speech delay**	**Motor delay**	**Epilepsy**	**Spasticity**	**Behavioral abnormalities**^**§**^	**Simplified gyration**	**Cortical thickening**	**Corpus callosum abnormalities**	**Pachygyria**	**Polymicrogyria**	**Schizencephaly**	**Heterotopia**		
Ex2	c.193G>A	p.V65M	Arab	7	yes	-5.3 to -9.8 SD	+ ^a,b^	+	+	-	+	-	+	-	+	-	+	+	-	disproportionate sized face and ears compared to the skull	[4,12]
Ex3	c.332G>C	p.R111T	Pakistani	6^#^		-7 to -11 SD		+	-	+	-	-									[11]
Ex4	c.363delT	p.D112fs	Mexican	3^#^	-5 SD	10 m: -5.4 SD		+	+	+	-	-	+	-	+	-	-	-	-		[12]
Ex5	c.535_536insA	p.M179fs	Indian	2^#^		-4 to -9 SD	+ ^a,c^	+	+	+	-	-	-	+	-	+	-	-	-	low set and prominent ears	[6]
Ex6	c.671G>C	p.W224S		3^#^		2y: -3.5 SD	+ ^c^	-	+	+	-	+	-	+	+	+	-	-	-	micrognathia	[7]
Ex8	c.900C>A	p.C300*	Indian	2^#^		-8 to -9 SD	+ ^a^	+	+	+	-	-	-	-	-	+	+	-	+		[6]
In8	c.1043 + 1G>A	p.S348fs	Turkish	1	-2.6 SD	2 m: -4.0SD		-	-	-	-	-	-	-	+	-	-	-	+		[12]
						5 m: -5.3SD															
Ex9	c.1143delA	p.H381fs	Pakistani	2^#^		-6 to -7 SD		+	+	+	-	-	-	-	+	-	-	+	-		[9]
Ex9	c.1194G>A	p.W398*	Pakistani	4^#^		-6 to -8 SD															[11]
Ex9	c.1198G>A	p.E400K	Hispanic	2^#^		9 m: -4 SD		+	+	+	-	+	-	-	-	+	-	-	-		[5]
Ex10	c.1313G>A	p.R438H	Pakistani	6	yes	-5 to -14 SD	+ ^a,b^	+	-	-	-	-	+	-	-	-	-	-	-	disproportionate sized face and ears compared to the skull	[4,8]
Ex10/23	c.1313G>A / c.2864-2867delACAG	p.R438H / p.D955fs	German	1	- 2.3 SD		+ ^a^	+	+	+	-	-	+	-	+	-	-	-	-	bilateral pes planus and hallux valgus	this study
Ex11	c.1408C>T	p.Q470*		1	yes		+ ^c^	-	-	+	-	-	-	+	+	+	-	-	-	genu varum, cryptorchidism, arachnodactly	[7]
Ex11	c.1531G>A	p.D511N	Pakistani	5	yes		+ ^a,b^	+	-	-	-	-								disproportionate sized face and ears compared to the skull	[4,8]
Ex12	c.1576G>T	p.E526*		1		9 m: -4 SD	+ ^b^	-	+	+	-	-	-	+	+	+	-	-	-		[7]
Ex12	c.1576G>A	p.E526K		1		3.5 y: -4 SD	+ ^c^	+	-	-	-	-	-	+	+	+	-	-	-	prognathism	[7]
Ex15	c.1942C>T	p.Q648*	Pakistani	2^#^	yes		+	-	-	-	-	-	+	-	-	-	-	-	-		[8]
Ex17/23	c.2083delA/c.2472_2473delAG	p.S696fs / p.Q918fs		2^#^	yes	8 y: -5 SD	+	+	+	+	+	-	-	-	-	-	+	-	-		[10]
In23	c.2867 + 4_c2867 + 7delGGTG	p.S956fs	Turkish	1		1 y: -8.5SD		-	+	-	-	-	-	-	+	-	-		+		[12]
Ex27	c.3232G>A	p.A1078T	Pakistani	5	yes		+ ^a,b^	+	-	-	-	-								disproportionate sized face and ears compared to the skull	[4]
Ex28	c.3361delG	p.A1121fs	Pakistani	2^#^		-10 to -11 SD															[11]
Ex29	c.3503G>A	p.W1168*	Pakistani	3^#^		-9 to -11 SD		-	-	+	-	+									[11]
Ex30	c.3839_3855del	p.G1280fs	Turkish	1		3 m: - 3.5SD		+	+	+	-	-	-	-	+	-	-	-	-		[12]
Ex30	c.3936dupC	p.V1314fs	Caucasian	5	-2.8 SD	-4 to -5 SD		+	+	-	-	-	+	+	+	-	+	-	-	broad nasal bridge, widely set eyes	[4,8,12]
			Turkish																		
			Pakistani																		
Ex31	c.4205_4208delTGCC	p.V1402fs	Turkish	2^#^		-5 to - 6 SD	+ ^c^	+	+	-	-	-	-	+	+	+	-	-	-	micrognathia, bulbous nose	[7]
Ex31	c.4241dupT	p.L1414fs	Pakistani	9	yes		+ ^a,b^	+		-	-	-								disproportionate sized face and ears compared to the skull	[4]

# Patients belong to one family.

* OFC in SDS, age in years (y) or months (m).

§ Behavioral abnormalities reported were hyperactivity and aggression.

a Mild, b Moderate, c Severe.

WDR62 is essential for mitotic spindle stabilization during mitosis and, as demonstrated in HeLa cells, it accumulates at the centrosome or the nucleus in a cell-cycle-dependent manner (from late prophase until metaphase-anaphase transition) [13]. It is enriched in proliferating precursors of the neuroepithelium in the developing murine brain [4,7] but at the same time it is also present in the cortical plate, a region where (postmitotic) neurons reside [12]. WDR62 knockdown in cortical progenitors by siRNA reduced their proliferative capacity, caused spindle orientation defects, decreased the integrity of centrosomes and displaced them from spindle poles, and delayed mitotic progression [13]. Despite considerable interest in MCPH as a model disorder for isolated and congenital microcephaly, the exact underlying pathomechanism remains to be established. Here, we report compound heterozygous mutations (one being novel) of the *WDR62* gene in a female MCPH2 patient of German descent and describe her clinical and cellular phenotype. We thereby provide evidence that the MCPH2 phenotype, at least partly, is due to centrosome/spindle organization defects.

### Human subjects and methods

#### Patients

Informed consent was obtained from the parents of the index patient for the publication of clinical and radiological data, cytogenetic and molecular genetic analyses, and lymphoblastoid cell line (LCL) studies. DNA was extracted from EDTA blood samples using standard techniques [14]. Approval to conduct the present study was obtained from the local ethics committee of the Charité (approval no. EA1/212/08). The index patient is a 24-year-old microcephalic patient of German descent with typical facial features of MCPH including sloping forehead and severe intellectual delay. She also had astatic seizures, which could be controlled by antiepileptic treatment. Cranial imaging studies revealed small frontal lobes, hypoplasia of the corpus callosum, simplified hippocampal gyration, widened lateral sulci, and cerebellar hypoplasia with an enlarged cisterna magna. Her blood count was normal, and there was no evidence of any malignant disease. The detailed phenotype is delineated below.

### Karyogram and array-CGH analysis

Standard karyotyping revealed a normal result (46,XX). Array-CGH was performed on the NimbleGen Whole Genome Tiling 135 k CGX-12 platform and revealed a 1.66 Mb duplication of the short arm of chromosome 2, arr[hg19] 2p12(82,018,317-83,674,828) × 3, that was inherited from the healthy mother and comprised a pseudogene (LOC1720) only.

### Exome sequencing

All three family members (parents, index patient) were subjected to exome sequencing. Genomic DNA was isolated from blood samples using standard methods. Five micrograms of genomic DNA were enriched using the Agilent Human All Exon V3 kit (Agilent Technologies, Santa Clara, CA, USA) following the manufacturer’s protocol. Whole-exome libraries were sequenced on an Illumina HiSeq 2000 system for 1 × 101 cycles following the manufacturer’s instructions (Illumina, San Diego, CA, USA). All raw sequencing reads were mapped onto UCSC hg19 [15] using Burrows-Wheeler Aligner (BWA) 0.5.9-r169 [16] and converted to BAM file format using SAMtools 0.1.18 [17]. Initial mappings were post-processed using the Genome Analysis Toolkit (GATK) 1.6 [18] following their ‘best practices V3′ (http://www.broadinstitute.org/gatk/guide/best-practices). In brief, reads were realigned around sites of known insertion-deletion polymorphisms (INDELs). Then, likely polymerase chain reaction PCR duplicates were detected using Picard 1.48 [17]. Finally, raw base quality scores were empirically recalibrated. Single nucleotide polymorphisms (SNPs) and INDELs were identified using the UnifiedGenotyper from GATK. Variants were classified as novel or known variants according to the SNP database (dbSNP) 135 [19]. Functional consequences of each variant were annotated using snpEff 2.0.5d20 [20] for UCSC hg19 RefSeq genes and ENSEMBL 65 human gene models [15,21]. The potential deleterious effect was evaluated using Polymorphism Phenotyping v2 (PolyPhen 2, [22]), sorting intolerant from tolerant (SIFT, [23]), PhyloP [24], MutationTaster [25], Genomic Evolutionary Rate Profiling (GERP++, [26]), Likelihood Ratio Test (LRT, [27]), and the OMIM database (http://www.ncbi.nlm.nih.gov/omim/), if available. Variants were filtered for the two most likely inheritance patterns, autosomal-dominant and autosomal-recessive (either compound heterozygous or homozygous).

### Sanger sequencing

The compound heterozygous mutations identified through exome sequencing were verified through Sanger sequencing using the primers: F1 5′-gtca tagtgctgtcattgagtcatc-3′, R1 5′gagccaactggcaaagaatc-3′, F2 5′-gtgccacacctcttcctcatc-3′, and R2 5′-cacctggaaccagggaacta-3′. The reference sequence NM_001083961 of *WDR62* was used.

### Establishment of Ebstein-Barr virus-transformed lymphocytes and culture

Ebstein-Barr virus-transformed lymphocytes (LCLs) were established according to the protocol published by H. Neitzel 1986 [28]. LCLs were cultured in RPMI 1640 with L-Glutamine (Invitrogen, Darmstadt, Germany) supplemented with 20% v/v fetal bovine serum (Invitrogen) and 1% v/v penicillin-streptomycin (Sigma-Aldrich, Taufkirchen, Germany) [29]. For this study, we used LCLs from the patient and from two controls.

### Immunocytology

For fixation, cells were cultured on poly-L-lysine (Sigma-Aldrich) coated coverslips for 30 min at standard conditions and subsequently incubated in paraformaldehyde (PFA) 4% for 10 min. Coverslips were rinsed with phosphate buffered saline (PBS 1x) and further incubated at room temperature in staining buffer (0.2% gelatin, 0.25% Triton X-100 in PBS 1x) for 20 min. Blocking was achieved by incubation in 10% donkey normal serum (DNS) in staining buffer for 30 min. Coverslips were incubated overnight at 4°C with primary antibodies in the staining buffer containing 10% DNS followed by an incubation with the corresponding secondary antibodies for 2 h at RT. Nuclei were labeled with 4′,6-diamidino-2-phenylindole (DAPI, 1:1,000, Sigma-Aldrich). Fluorescently labeled cells were analyzed and imaged by a fluorescent Olympus BX51 microscope with the software Magnafire 2.1B (version 2001; Olympus, Hamburg, Germany), and all images were processed using Adobe Photoshop. This procedure has been previously described [29].

The anti-WDR62 antibody (rabbit polyclonal anti-WDR62, Bethyl laboratories, A301-560A, 1:500) utilized in this study recognizes amino acids between residue 900 and 950 of human WD repeat domain 62 (accession no. NP_775907.4, GeneID 284403, UniProt ID: O43379). Further primary antibodies were as follows: mouse anti-γ-tubulin (T6557, Sigma-Aldrich, 1:500), mouse anti-α-tubulin (T9026, Sigma-Aldrich; 1:1,500), and rabbit anti-CDK5RAP2 (HPA035820, Sigma-Aldrich, 1:200). The immunoreaction specificity was analyzed in control specimen incubated only in the secondary antibodies.

### Protein extraction procedure and Western blot

Protein extracts for Western blots were isolated from LCLs by homogenization in radio-immunoprecipitation assay (RIPA) buffer containing 1 mM phenylmethylsulfonyl fluoride (PMSF; Sigma-Aldrich) and 1 protease inhibitor cocktail tablet per 10 ml RIPA buffer (Complete Mini; Roche Diagnostics, Mannheim, Germany), 15 min incubation on ice, followed by ultrasonication and centrifugation at 4°C for 10 min at 3,000 g and for 20 min at 16,000 g. Protein concentrations were determined using a bicinchoninic acid (BCA) based assay, according to the instructions of the manufacturer (BCA Protein Assay Kit; Pierce Biotechnology, Rockford, IL, USA). Protein extracts (30 μg per sample) were denaturated in Laemmli sample loading buffer at 95°C for 5 min, separated by sodium dodecyl sulphate polyacrylamide gel electrophoresis (SDS-PAGE), and electrophoretically transferred onto nitrocellulose membrane (Bio-Rad, Munich, Germany) using Bio-Rad wet transfer system (Bio-Rad, Munich, Germany). This procedure has been previously described [29].

The membranes were incubated for 1 h at room temperature in blocking buffer Tris-Buffered Saline Tween-20 (TBS-T) 1× with 5% bovine serum albumin (BSA), rinsed three times with TBS-T (1×) for 8 min each at RT on a shaker, and then incubated overnight at 4°C with rabbit anti-WDR62 (1:500, A301-560A, Bethyl laboratories) or mouse anti-actin (1:10,000, MAB1501, Millipore) antibodies in blocking solution. After incubation with the corresponding secondary antibodies donkey anti-rabbit (1:2,000; Amersham Biosciences, Freiburg, Germany) and goat anti-mouse (1:10,000; Dako, Hamburg, Germany) the immunoreactive proteins were visualized using a technique based on a chemiluminescent reaction. The gel pictures were obtained using photographic films (Amersham Hyperfilm ECL, GE Healthcare, UK). Western blot experiments were run in triplicate.

### Cell cycle analysis

Cell cycle analysis was performed using the 5-bromo-2′-deoxyuridine (BrdU)-Hoechst 33258 method [30]. The principle of that assay is based on the incorporation of the halogenated base analog during DNA replication. The assay makes use of the fact that BrdU-substituted chromatin quenches the fluorescence of the dye Hoechst 33258. This method differentiates not only between cycling and noncycling cells in a given culture but also recognizes the distribution of the cycling cells in as many as four consecutive cycles. For flow cytometry mononuclear cells were Ficoll-isolated from heparinized blood samples. Cultures were set up in RPMI 1640 medium with GlutaMAX (Gibco Life Technologies), supplemented with 15% fetal bovine serum (FBS, PAN Biotech). Duplicate cultures were either left untreated, or irradiated with 1.5 Gy from a linear accelerator or continuously exposed to 10 ng/ml mitomycin C (Medac). To all of the cultures BrdU (Sigma-Aldrich) was added at a final concentration of 10^-4^ M [31]. Lymphocyte growth activation was induced by phytohaemagglutinin (PHA HA16, Remel Europe, Dartford, UK). The cells were harvested after 72 h. Following staining with Hoechst 33258 (1.2 μg/ml; Molecular Probes) for a minimum of 15 min in buffer containing 154 mM sodium chloride (NaCl), 0.1 M TRIS pH 7.4, 1 mM calcium chloride (CaCl_2_), 0.5 mM magnesium chloride (MgCl_2_), 0.2% BSA, and 0.1% nonyl phenoxypolyethoxylethanol (NP40) in distilled water in the dark, ethidium bromide (EB, 1.5 μg/ml, Molecular Probes) was added for another minimum of 15 min. Bivariate flow histograms were recorded on a triple-laser equipped LSRII flow cytometer (Becton Dickinson) using UV excitation of Hoechst 33258 and 488-nm excitation of EB. The resulting cell cycle distributions reflecting cellular DNA content were quantified using the MPLUS AV software package (Phoenix Flow Systems, San Diego, CA, USA).

### Analysis of chromosome condensation

Whole-blood cultures were prepared using RPMI 1640 medium with GlutaMAX (Gibco Life Technologies) supplemented with 15% fetal bovine serum (FBS, PAN Biotech). Lymphocytes were stimulated with PHA (Remel Europe). The cultures were terminated after 72 h following addition of 8 μl/ml Colcemid solution (10 μg/ml; PAA) for the final 45 min. Metaphase preparations were made by hypotonic incubation of the cell pellets (0.075 M KCl for 10 min at 37°C) and fixation of the nuclei in ice-cold methanol/concentrated acetic acid 3:1. Slides were stained with 5% Giemsa solution for 5 min without applying banding techniques. A total 1,000 nuclei per sample were scored by visual counts, and the proportion of metaphases and of nuclei with prophase-like morphology (prophase-like cells, PLCs, defined as nucleus-shaped structures with condensed chromosomes) were determined.

## Results

### Phenotype of the patient with MCPH2

The index patient is the third child of non-consanguineous healthy parents of German descent (Figure 1A). Microcephaly was diagnosed by ultrasound in the 30th week of gestation. Pregnancy and delivery were uneventful. Birth weight, length, and head circumference at term were 3600 g (0.6 SDS, 50-75th centile), 55 cm (1.6 SDS, 95th centile), and 31 cm (-2.3 SDS; 1 cm below 3rd centile), respectively. The head circumference of her parents was normal. Postnatal cranial ultrasound revealed small frontal lobes, hypoplasia of the corpus callosum, simplified hippocampal gyration, widened lateral sulci, and cerebellar hypoplasia with an enlarged cisterna magna. Further imaging studies were refused by the parents. These findings are in line with cortical malformations previously associated with *WDR62* mutations, and are expected to have impact on cognitive, language, motor, and behavioral functions of the patient. Language development of the girl was severely delayed (first words at the age of 4 years) while the delay in motor development (unaided walking at the age of 2 years) was moderate [32]. At the age of 16 years, the patient developed astatic seizures that were controlled by carbamazepine treatment. At the age of 24 years, the patient was distinctively microcephalic (50 cm, 3 cm below 3rd centile, -3.3 SDS) while her body length of 164 cm (25th-50th centile) and weight of 54 kg (25th-50th centile) were normal. She could phrase sentences of up to three to four words and recognized letters but was not able to perform any abstract intellectual task such as reading, writing or simple calculation. She also had difficulties in performing complex motor tasks such as riding a bicycle. Neurological examination did not reveal any further abnormality. Spatial orientation, vision and hearing were normal. Facial features included convex facial profile, sloping forehead, marginally low-set and posteriorly rotated ears, small chin, and full lips. In spite of the small cranium, palpebral fissures were horizontal. On physical examination bilateral pes planus and hallux valgus were found. Blood count was normal, and there was no evidence of any malignancy or further organ malformation/malfunction (Table 1). Results of genetic analyses (karyogram and array-CGH) were normal.

The patient’s sister who was born earlier also had microcephaly and intellectual disability (Additional file 1: Figure S1). She died of a Wilms tumor at the age of 5 years (head circumference at birth 31 cm, -2.3 SDS, 1 cm below 3rd centile; head circumference at 2 years-of-age 41 cm, -4.4 SDS, 5 cm below 3rd centile). A left-sided spastic hemiparesis had been noticed. Brain computed tomography at one month of age had revealed a plump right ventricle, wide arachnoidal spaces, and parieto-occipital hypodensity. In the second year of life, an electroencephalogram (EEG) had revealed signal depression on the right hemisphere and epileptic activity on the left hemisphere. Results of genetic analyses (Karyogram and array-CGH) had been normal.

### Novel *WDR62* mutation

Both parents and the index patient were subjected to whole exome sequencing. In total, we obtained 92–100 million single-end 101 bp reads per sample, of which 97.6 - 98.0% could be mapped onto the human genome. After removing duplicated reads, which were possibly derived from PCR artifacts, 29–31 million unique reads were mapped to the targeted protein coding regions, resulting in an average of 59.0 - 62.8× coverage within the targeted coding region. Using the Genome Analysis Toolkit (GATK) we detected 19,381 - 19,656 SNPs and INDELs in the exome of each family member, of which 98.3 - 98.4% were known variants deposited in dbSNP 135. Given the pedigree, we searched for autosomal-dominant, autosomal-recessive homozygous, compound heterozygous, and de novo mutations.

**Figure 2:**
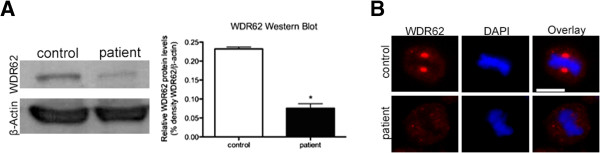
**Representation of WDR62 protein in immortalized lymphocytes. (A)** Protein extracts were analyzed with an anti-WDR62 antibody that recognizes amino acids 900–950. Beta-actin was used as a control for equal protein loading. Signals were visualized with enhanced chemiluminescence. WDR62 protein levels in immortalized lymphocytes from the patient were significantly lower than in control cells (n = 3, p 0.0104, Student’s t-test). **(B)** Centrosomal WDR62 levels were below detection levels when assessed through immunocytology in patient cells using the same antibody.

Through this exome sequencing approach, we identified two compound heterozygous mutations of the *WDR62* gene in the index patient: (i) a missense mutation (c.1313G>A) in exon 10 that was inherited from the mother and resulted in a substitution of arginine by histidine (R438H); (ii) a frameshift mutation with deletion of 4 nucleotides (c.2864-2867delACAG) in exon 23 that was inherited from the father and resulted in a stop codon of the new reading frame 112 aa downstream of the deletion (D955Afs*112). Both mutations were confirmed by Sanger sequencing (Figure 1C). The *WDR62* gene encodes for a WD-repeat containing protein and has been associated with autosomal recessive primary microcephaly 2 (MCPH2, MIM*613583), matching the phenotype of the patient. c.1313G>A has been described previously in seven homozygous MCPH2 patients and is predicted to be deleterious [4], while c.2864-2867delACAG is a novel mutation and could result in nonsense-mediated decay. Indeed, WDR62 protein levels were reduced or below detection level in EBV-transformed lymphocytes of the index patient, when assessed via western blot and immunocytology, respectively (Figure 2). A survey of the types and locations of reported *WDR62* mutations is schematically depicted in (Figure 1E). Independent of the two mutations, the patient also carried a 1.66 Mb duplication at 2p12 (chr2: 82.0-83.7 Mb, hg 19). The duplication comprising the pseudogene LOC1720 was inherited from her healthy mother and thus was not considered as disease-causing.

### Cellular phenotype of the patient with *WDR62* gene mutations

**Figure 3:**
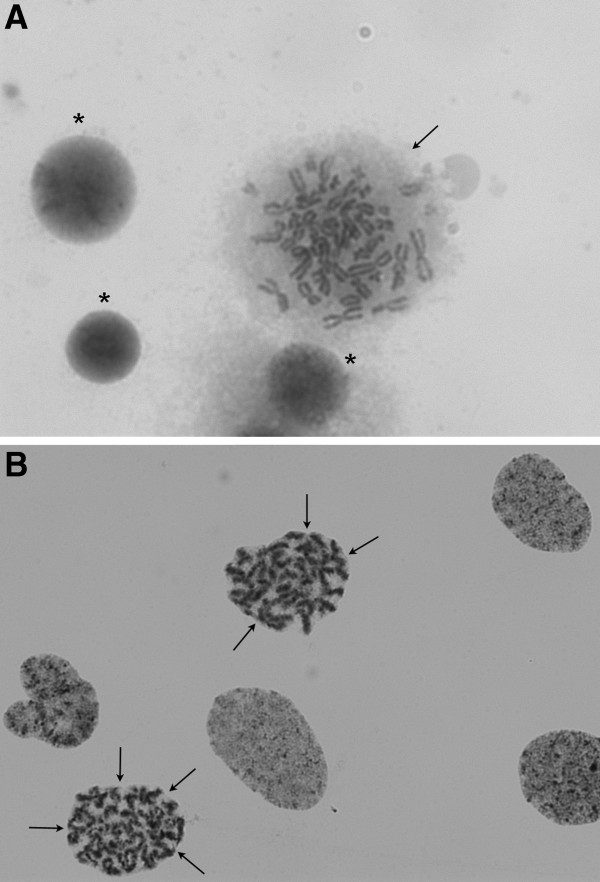
**Absence of a prophase-like chromosome phenotype in MCPH2 deficiency. (A)** Normal metaphase morphology (arrow) of a cultured lymphoblast in a cell line from the MCPH2 patient after induced mitosic arrest by colcemid. The chromosomes are condensed while the delimitation of the metaphase is irregularly shaped suggesting that there is no more nuclear membrane after its disintegration in prophase. Three other nuclei are stained rather homogeneously, typical of uncondensed chromatin in interphase nuclei (asterisks). **(B)** An MCPH1 lymphoblastoid cell line treated identically shows nuclei with typical prophase-like chromosome (PLC) morphology. Virtually all of the six nuclei reveal a meandering striped or banded chromatin structure characteristic of beginning chromosome condensation (best seen in the two marked nuclei). Despite prometaphase stage, they present with rounded delimitations (arrows) suggesting that they are bounded by a persisting nuclear membrane, a phenomenon that is designated as premature chromosome condensation (PCC). Giemsa stain.

We assessed the phenotypic consequences of the biallelic WDR62 mutation in mutant lymphoblastoid cells (LCLs) of the MCPH2 patient. Cell cycle analysis of LCLs revealed normal results when compared to control specimen, and the patient’s LCLs did not prove sensitive to ionizing radiation or mitomycin C exposure (data not shown). After colcemid arrest of otherwise untreated cultures, a vast majority of mitotic cells in the patient’s LCLs showed typical metaphase morphology (Figure 3A) in contrast to MCPH1 cells whose characteristic feature is an increased number of nuclei with prophase-like chromosome (PLC) morphology (Figure 3B). Quantitative analysis revealed a regular PLC rate of the MCPH2 LCLs, comparable to normal controls and opposed to the increased rates in MCPH1 LCLs (Table 2).

**Table 2:** Rate of nuclei with prophase-like chromosome (PLC) morphology

**Designation**	**Mean [%]**	**PLC Rate SD [%]**	**Range [%]**	**n**
MCPH2 Patient	1.36	± 0.5	0.79-2.33	7
Normal Controls	1.17	± 0.85	0-2.09	8
MCPH1 Patients	9.07	± 4.02	5.96-13.77	3

N: number of independent experiments in the reported MCPH2 patient or number of individuals with single experiments in normal and MCPH1 controls.

**Figure 4:**
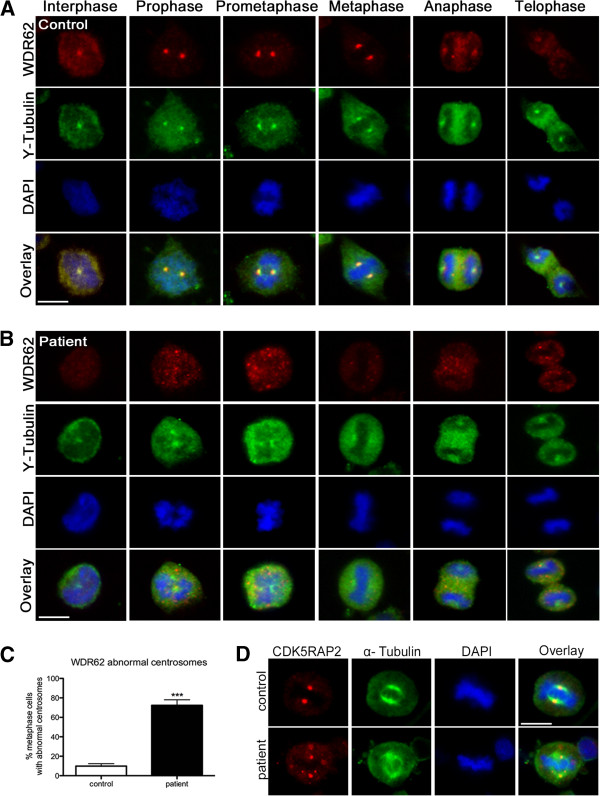
**WDR62 in immortalized lymphocytes and dispersion of centrosomal proteins γ-tubulin and CDK5RAP2 in *WDR62 *mutant patients cells.** Subcellular localization of WDR62 (red) and γ-tubulin (green) throughout the cell cycle in immortalized lymphocytes of **(A)** controls and **(B)** the MCPH2 patient. In controls, WDR62 colocalized with γ-tubulin and was present on the centrosome with high levels throughout mitosis until anaphase and telophase, thereafter the signal intensity dropped to interphase levels. Gamma-tubulin immunostaining shows distinct centrosomes in controls whereas in patient cells the γ-tubulin-marked centrosomes appear broad and dispersed. **(C)** Quantification results of abnormal centrosomes with a dispersed γ-tubulin staining around the centrosome (n = 100 metaphase lymphoblastoid cells, p = 0.0006, Student’s t-test). **(D)** Abnormal localization of centrosomal protein CDK5RAP2 (red) in *WDR62* mutant patient immortalized lymphocytes (see also Figure 5). Cells were stained for WDR62, for the centrosome marker CDK5RAP2, and for α-tubulin as a microtubuli marker. DNA was stained with DAPI (blue).

We determined the subcellular distribution of WDR62 in LCLs of patient and controls by immunocytology. In control cells, WDR62 localized to the centrosomes throughout the mitotic progression with reduced signal intensities in anaphase and telophase (Figure 4A). Centrosomal WDR62 signals were relatively reduced during interphase and then increased throughout mitosis until anaphase, when signals dropped to interphase levels. In anaphase telophase, WDR62 also appeared to be present in the division plane/cytoplasm. Furthermore, WDR62 was assigned to the midbody during anaphase as shown by co-localization with γ-tubulin (Figure 4A) for which midbody localization has been reported [33]. In *WDR62* mutant cells the co-localization of WDR62 and γ-tubulin at the midbody appeared to be abrogated to a large extent similar to the reduced levels at the centrosomes (Figure 4B). In *WDR62* mutant LCLs, WDR62 expression was not detectable when assessed by immunocytology using an antibody against a WDR62 epitope at aa 900 to 950 (Figure 2B, Figure 4B). Western blot using the same antibody revealed a single band of normal size but severely reduced quantity, suggesting that the allele with the frameshift deletion was not expressed (otherwise a second band would be visible) while the allele with the missense mutation had residual expression and/or enhanced degradation and possibly a more dispersed distribution making its immunocytological detection impossible (Figure 2A).

**Figure 5:**
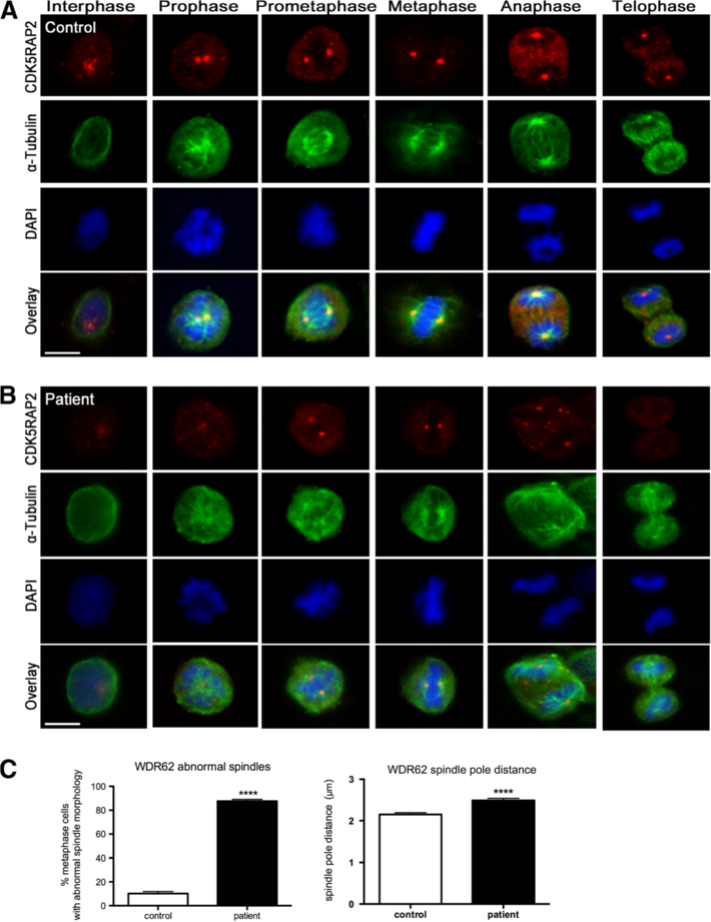
**Spindle defects in *WDR62 *mutant patient cells.** Subcellular localization of CDK5RAP2 and α-tubulin throughout the cell cycle in immortalized lymphocytes of **(A)** control and **(B)** MCPH2 patient. While the spindle apparatus has a regular bipolar form of appearance from prometaphase to telophase in controls, patient cells show abnormal spindle formation with an increase of abnormal misdirected spindles and broad, unfocused microtubules poles. CDK5RAP2 signals are weaker in patient’s cells than in controls. Cells were stained for CDK5RAP2 (red), for α-tubulin (green), and for DNA using DAPI (blue). **(C)** Quantification results of abnormal spindles and spindle pole distance (n = 115 metaphase lymphoblastoid cells, p < 0.0001, Student’s t-test).

Since deficiency or dysfunction of WDR62 reduce human brain size and impact on cell proliferation, we examined the integrity of the centrosome and the mitotic spindle apparatus in cells of the index patient and of the controls. In control LCLs, WDR62 colocalized with the centrosomal protein γ-tubulin throughout the cell cycle (Figure 4A). In patient cells, WDR62 was below the detection limit. Here, we observed a more dispersed γ-tubulin staining around the centrosome rather than a complete loss of γ-tubulin from the centrosome (72.3% versus 9.8% of 100 counted metaphase lymphoblastoid cells of index patient versus controls; Student’s t-test, p < 0.001) (Figure 4B, C). Similarly, the levels of centrosomally located CDK5RAP2 were strongly reduced in *WDR62* mutant LCLs (Figure 4D, Figure 5A,B).

**Figure 6:**
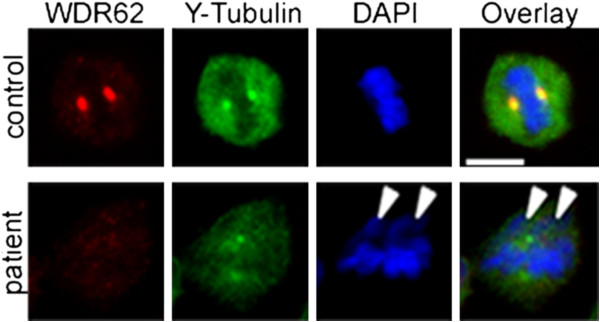
**Lagging chromosomes in mutant *WDR62 *lymphocytes.** Some chromosomes in patient cells showed lagging defects in metaphase (indicated by arrows).

We further examined the changes in mitotic spindle organization. In controls, the spindle apparatus had a regular bipolar form of appearance from prometaphase to telophase (Figure 5A). Spindle defects were observed in patient cells with an increase of abnormal misdirected spindles with broad and unfocused poles of microtubules (Figure 5B). Quantification of the metaphase cell population indicated that more than half of WDR62-depleted cells exhibited such abnormal bipolar spindles (87.6% versus 10.2% of 115 counted metaphase LCLs of index patient versus controls; Student’s t-test, p < 0.0001) (Figure 5C). Mutant cells also showed displaced centrosomes detected by co-staining with both CDK5RAP2 and alpha-tubulin (Figure 4D). In addition, the spindle pole distance was significantly increased in mutant cells compared to controls (2.46 μm versus 2.1 μm of 115 counted metaphase lymphoblastoid cells of index patient versus controls; Student’s t-test, p < 0.0001) (Figure 5C). Given the importance of assembly of bipolar mitotic spindles for accurate chromosome segregation, we investigated the alignment of chromosomes in patient cells. We noted the presence of lagging chromosomes in some lymphoblastoid cells from the patient compared to the controls (Figure 6).

## Discussion

### Phenotype and genotype

We report compound heterozygous *WDR62* mutations in a German girl with primary microcephaly: (i) a missense mutation with single nucleotide transition c.1313G>A in exon 10 resulting in the substitution of arginine by histidine (R438H) which has been reported previously in homozygous patients (4), and (ii) a novel frameshift deletion of four nucleotides c.2864-2867delACAG in exon 23 that resulted in a stop codon of the new reading frame 112 aa downstream of the deletion (p.D955Afs*112) (Figure 1). The index patient had congenital microcephaly, intellectual disability, speech deficit, and epilepsy. Apart from typical facial features of MCPH including a sloping forehead, she had only minor dysmorphic features (convex profile with small chin), and her height and weight were normal. There was no further neurological deficit and her sensorial functions were normal. The postnatal cranial ultrasound of the patient revealed small frontal lobes, simplified hippocampal gyration, and hypoplasia of both the corpus callosum and the cerebellum. Despite the classical definition of MCPH as a severe congenital microcephaly lacking morphological abnormalities of the brain, it is now acknowledged that particularly MCPH patients with *WDR62* mutations may have a wide spectrum of brain malformations in addition to microcephaly including pachygyria, thickened cortex, polymicrogyria, schizencephaly, corpus callosum, and hippocampal abnormalities as well as cerebellar hypoplasia [4,7,12], in line with the phenotype of the index patient (Table 1).

Although cancer has not been described so far in patients with MCPH2, individual patients with other MCPH subtypes and leukemia have been reported [2,34]. Moreover, mouse models of MCPH3 and MCPH5 displayed an increased tumor risk and/or blood abnormalities (anemia, leucopenia) [35,36]. It is likely that the deceased sister of the index patient who also had severe microcephaly at birth and intellectual disability, carried the p.R438H / p.D955Afs*112 mutations of the *WDR62* gene. (Unfortunately, her DNA was not available for genetic testing anymore.) The diagnosis of a Wilms tumor in association with a putative *WDR62* mutation raises concerns regarding a potentially increased cancer risk in MCPH2 patients also. In addition, especially in patients with MCPH1 and MCPH5, early puberty, renal agenesis, and multicystic kidneys have been described. As this point warrants further investigation in patients, we investigated the clinical phenotype of our patient in detail but found no evidence of blood abnormalities, organ involvement, or malignancy.

### Effect of *WDR62* mutations

Most previously reported *WDR62* mutations have been proposed to lead to loss of WDR62 function (Figure 1, Table 1). The mutation of the paternal allele (p.D955Afs*112) in the present patient leads to a stop codon in the novel reading frame. Since the Western blot revealed only a single band of normal size we assume that this allele’s RNA is subject to nonsense-mediated decay and that its protein product is not expressed (Figure 2A). The previously reported missense mutation present also in our index patient (p.R438H) alters evolutionarily highly conserved amino acids of WDR62 [4].

The human MCPH phenotype is considered, based on results from *in vivo* and *in vitro* studies, to result from a premature shift from symmetric to asymmetric neural progenitor-cell divisions (with a subsequent depletion of the progenitor pool) and from a reduction in cell survival [37,38]. A central molecular mechanism for microcephaly in MCPH2 may be a deficiency or dysfunction of WDR62 at the spindle pole of dividing cells due to processes such as non-expression, loss of essential spindle targeting domains, misfolding or rapid degradation of the mutant protein [39]. For WDR62 it has been recently demonstrated that siRNA downregulation in murine neural progenitors through *in utero* electroporation induces early cell cycle exit and a reduced proliferative capacity. Downregulation by siRNA of *WDR62* in murine neural progenitors causes early cell cycle exit and reduced proliferative capacity [13]. Moreover, WDR62 downregulation in tumor cells (HeLa) was associated with spindle orientation defects, decreased centrosome integrity and displacement from the spindle pole as well as delayed mitotic progression [13].

### Cellular phenotype

To study the effect of the reported compound heterozygous *WDR62* gene mutations on centrosome and spindle integrity in the present index patient, we analyzed the intracellular localization of WDR62 as well as centrosome and spindle morphology of EBV-transformed lymphocytes (LCLs) from the patient and from controls. WDR62 localized to the centrosomes of control LCLs at all phases of the cell cycle in a cycle-dependent manner, with low centrosomal WDR62 levels in anaphase and telophase. In interphase, WDR62 was also only weakly associated with the γ-tubulin-labeled centrosome; moreover, WDR62 expression appeared to be cytosolic (Figure 4). These findings are in line with previous reports of WDR62 localization in human tumor cells (HeLa, HEK293, A549, HepG2), human non-tumor cells including B-lymphocytes as well as mouse cerebral cortex neuroepithelial cells at E13. Here WDR62 was reported to assume both nuclear and cytosolic distribution in interphase by some authors [4,6,12,13], while accumulating strongly at the spindle poles during mitosis [4,6,12,13] with no obvious centrosomal association of WDR62 during anaphase and telophase [13]. We also detected WDR62 protein accumulation in the division plane (midbody) in lymphoblastoid cell lines, a region of bipolar microtubuli array that arranges between separating sister chromatids during anaphase [40]. Contrary to our results, no protein accumulation was detected at the midbody during cytokinesis in previous localization reports performed in other cell lines (HeLa, HEK293, A549, HepG2) [4,6,12]. Further studies are needed to address the association of WDR62 with the midbody. In one report, the mitotic WDR62 distribution was evaluated by its colocalization with CDK5RAP2, a key centrosomal protein known to cause MCPH3 when disrupted. WDR62 and CDK5RAP2 colocalized to the centrosome throughout the cell cycle. However, during prometaphase and metaphase, WDR62 surrounded rather than strictly colocalized with CDK5RAP2 at the centrosome [7].

WDR62 protein levels were low (western blot) or non-detectable (immunocytology) in MCPH2 patient cells. As indicated above, this finding may result from nonsense-mediated decay of the transcript with the frameshift deletion and dispersion and/or instability of the protein with the missense mutation (Figures 2 and 4). In contrast to siRNA studies on *WDR62* downregulation, we detected neither a disorder of cell cycle progression in peripheral lymphocytes of the patient nor increased chromosomal breakage rates of her LCLs when compared to controls. We could, however, detect a failure of the centrosomal proteins γ-tubulin and CDK5RAP2 to localize properly at the centrosome in patient cells indicating an abnormal centrosome integrity (Figures 4 and 5). In addition, spindles were disorganized in patient cells with displaced centrosomes, increased spindle pole distance, and lagging chromosomes (Figures 5 and 6). These results are in line with those of Bogoyevitch et al. 2012 [13] who observed abnormal metaphase spindles characterized by a displacement of centrosomes from the spindle pole and a significant increase of spindle length. Thus, the recent appreciation of a relationship between proper centrosome attachment to the spindle pole and spindle length determination [41,42] is reinforced. Since spindle function is important for correct chromosome alignment at the spindle equator and this again is necessary for accurate segregation of chromosomes during cell division into two daughter cells [43], we investigated these processes. Our observation of disturbed metaphase chromosome alignment and of lagging chromosomes in some lymphoblastoid cells from the patient is in line with previous reports demonstrating an increase in the proportion of unaligned chromosomal DNA during metaphase in HeLa cells following *WDR62* siRNA [13]. This is consistent with delayed mitotic progression and disrupted centrosome/bipolar spindle organization [13], emphasizing the importance of regulated assembly of the mitotic spindle apparatus that impact the forces acting on chromosomes.

## Conclusion

Tight and timely control of cell division largely determines brain size during embryonic development [44]. Our results, although generated in human patient lymphocytes and not human neural progenitors, suggest that spindle defects and a disruption of centrosome integrity could play a role in the development of microcephaly in MCPH2. In fact, our results on lymphoblastoid cells from a MCPH2 patient are in line with those detected in two MCPH5 patients [29]. Future studies on neuronal stem cells derived from iPS cells of MCPH patients may provide further confirmation.

## Abbreviations

Array-CGH: Comparative genomic hybridization; BCA: Bicinchoninic acid; BrdU: 5-bromo-2-deoxyuridine; BSA: Bovine serum albumin; BWA: Burrows-wheeler aligner; CaCl2: Calcium chloride; CDK5RAP2: CDK5 regulatory subunit associated protein 2; DAPI: 4′,6-diamidino-2-phenylindole; dbSNP: Single nucleotide polymorphism database; DNS: Donkey normal serum; EB: Ethidium bromide; EBV: Epstein–Barr virus; EDTA: Ethylene diamine tetraacetic acid; EEG: Electroencephalogram; FBS: Fetal bovine serum; GATK: Genome analysis toolkit; GERP++: Genomic evolutionary rate profiling; INDELs: Insertion-deletion polymorphisms; LCLs: Lymphoblastoid cell lines; LRT: Likelihood ratio test; MCPH: Autosomal recessive primary microcephaly; MgCl2: Magnesium chloride; NaCl: Sodium chloride; NP40: Nonyl phenoxypolyethoxylethanol; PBS: Phosphate buffered saline; PCR: Polymerase chain reaction; PFA: Paraformaldehyde; PHA: Phytohaemagglutinin; PLCs: Prophase-like cells; PMSF: Phenylmethylsulfonyl fluoride; PolyPhen 2: Polymorphism phenotyping v2; RIPA: Radio-immunoprecipitation assay; SDS-PAGE: Sodium dodecyl sulphate polyacrylamide gel electrophoresis; SIFT: Sorting intolerant from tolerant; siRNA: Small interfering RNA; SNPs: Single nucleotide polymorphisms; TBS-T: Tris-buffered saline tween-20; WDR62: WD repeat-containing protein 62.

## Competing interests

The authors declare no competing interest in the preparation or publication of the data in this manuscript.

## Authors’ contributions

AMK, KO, and WC were responsible for the project conception. AMK and HGF wrote the manuscript. KO and AMK attended the patients and provided clinical data. AH established the LCLs culture. HGF, ER, and NK performed the lymphocyte analysis, performed Sanger sequencing, and generated figures. SF and WC performed exome sequencing and data analysis. TS assessed PCL rates. DS and TS performed cell cycle and chromosome breakage analysis and revised the manuscript. All authors read and approved the final manuscript.

## Supplementary Material

Additional file 1: Figure S1 Picture of sibling. Picture of the patient’s sister (II:1, see pedigree in Figure 1A) at the age of 1.5 years. She also had microcephaly and intellectual disability, and she died of a Wilms tumor at the age of 5 years. Click here for file
