# Supplementary figures and images for: Abnormal centrosome and spindle morphology in a patient with autosomal recessive primary microcephaly type 2 due to compound heterozygous WDR62 gene mutation
Source: Orphanet J Rare Dis. 2013 Nov 14;8:178. doi: 10.1186/1750-1172-8-178 (PMC4225825; doi:10.1186/1750-1172-8-178)

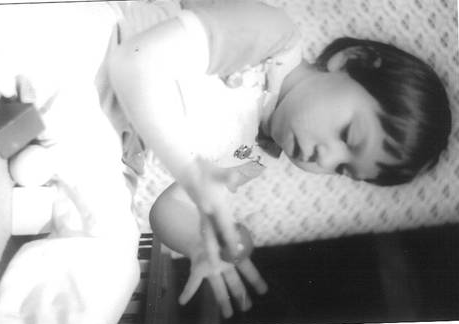

Supplement: Additional file 1: Figure S1 — Picture of sibling. Picture of the patient’s sister (II:1, see pedigree in Figure 1A) at the age of 1.5 years. She also had microcephaly and intellectual disability, and she died of a Wilms tumor at the age of 5 years. [file 1750-1172-8-178-S1.tiff]
